# A Web-Based Dynamic Nomogram to Predict the Risk of Methicillin-Resistant Staphylococcal Infection in Patients with Pneumonia

**DOI:** 10.3390/diagnostics14060633

**Published:** 2024-03-16

**Authors:** Van Duong-Thi-Thanh, Binh Truong-Quang, Phu Tran-Nguyen-Trong, Mai Le-Phuong, Phu Truong-Thien, Dung Lam-Quoc, Thong Dang-Vu, Minh-Loi Nguyen, Vu Le-Thuong

**Affiliations:** 1 Faculty of Medicine, University of Medicine and Pharmacy, Ho Chi Minh 700000, Vietnam; dttvan@ctump.edu.vn; 2 Faculty of Medicine, Can Tho University of Medicine and Pharmacy, Can Tho 900000, Vietnam; tntphu@ctump.edu.vn; 3 Department of Cardiology, University Medical Center, Ho Chi Minh 700000, Vietnam; 4 Faculty of Medicine, Chulalongkorn University, Bangkok 10330, Thailand; 5 Department of Microbiology, Cho Ray Hospital, Ho Chi Minh 700000, Vietnam; phuongmaimdcr@gmail.com (M.L.-P.); truongthienphu78@yahoo.com (P.T.-T.); 6 Department of Pulmonary, Cho Ray Hospital, Ho Chi Minh 700000, Vietnam; lamquocdung69@gmail.com (D.L.-Q.); drthongdvu@gmail.com (T.D.-V.); 7 Faculty of Information Technology, Ho Chi Minh City University of Science, Ho Chi Minh 700000, Vietnam; 22120189@student.hcmus.edu.vn; 8 Department of Pulmonary, University Medical Center, Ho Chi Minh 700000, Vietnam

**Keywords:** pneumonia, methicillin resistance, *Staphylococcus*, risk factors, nomograms

## Abstract

The aim of this study was to create a dynamic web-based tool to predict the risks of methicillin-resistant *Staphylococcus* spp. (MRS) infection in patients with pneumonia. We conducted an observational study of patients with pneumonia at Cho Ray Hospital from March 2021 to March 2023. The Bayesian model averaging method and stepwise selection were applied to identify different sets of independent predictors. The final model was internally validated using the bootstrap method. We used receiver operator characteristic (ROC) curve, calibration, and decision curve analyses to assess the nomogram model’s predictive performance. Based on the American Thoracic Society, British Thoracic Society recommendations, and our data, we developed a model with significant risk factors, including tracheostomies or endotracheal tubes, skin infections, pleural effusions, and pneumatoceles, and used 0.3 as the optimal cut-off point. ROC curve analysis indicated an area under the curve of 0.7 (0.63–0.77) in the dataset and 0.71 (0.64–0.78) in 1000 bootstrap samples, with sensitivities of 92.39% and 91.11%, respectively. Calibration analysis demonstrated good agreement between the observed and predicted probability curves. When the threshold is above 0.3, we recommend empiric antibiotic therapy for MRS. The web-based dynamic interface also makes our model easier to use.

## 1. Introduction

Methicillin-resistant *Staphylococcus* spp. (MRS) is an important pathogen in pneumonia [1,2]. Historically, methicillin-resistant *Staphylococcus aureus* (MRSA) was designated a pneumonia pathogen with varying prevalence across countries [3]. Since 2022, methicillin-resistant coagulase-negative *Staphylococcus* spp. (MRCoNS) has also been considered a pathogen in pneumonia [2]. MRS infections in pneumonia were associated with increased total hospital costs, a prolonged hospital stay, and poor outcomes [4,5]. More significantly, MRS infection is linked to an 18.7–40.8% mortality rate across Asian countries [6].

The risk of MRS infection in pneumonia needs to be addressed when designing empirical therapy regimens. According to previous studies, risk factors for MRS infection included tobacco use, chronic obstructive pulmonary disease, recent antibiotic exposure, illicit drug use, and chest tubes [2,7]. MRSA pneumonia was also associated with male gender, age over 74, diabetes, a recent nursing home or hospital stay, recent exposure to fluoroquinolones or antibiotics for Gram-positive organisms, and severe pneumonia [8]. Therefore, clinical settings need to be based on these risk factors to cover MRS early to improve prognosis in these patients.

Coverage treatment based on guidelines helps provide early MRS treatment; however, related risk factors need to be considered more. The American Thoracic Society (ATS) and the British Thoracic Society (BTS) both made suggestions about risk factors for treating MRSA. These included having used an intravenous antibiotic in the past 90 days, having severe pneumonia, having been infected with MRSA before, being treated in units with more than 10–20% MRSA isolates [9], having a shadow on both lungs, and having frequent lung cavitation [10]. The goals of the guidelines help improve targeted treatment for MRSA; however, the frequency of MRSA and MRCoNS infection and the outcome have been the topic of much discussion. Several studies have been developed from these recommendations to identify additional risk factors beyond those already included in existing guidelines to better identify patients at higher risk, which could help tailor treatment decisions more precisely, reducing the risk of overtreatment.

Nomograms are graph-based tools used to calculate the predicted probability of an event of interest using the input parameters. Compared to traditional forms, web-based dynamic nomograms are gaining widespread use in prognosis and diagnosis of pulmonology and other disciplines thanks to their user-friendliness and accessibility [11,12]. The application simplifies the process by integrating the underlying regression formula of the prediction model into its calculation, thereby enabling rapid results for predicted risk. Its convenience may allow for tailored clinical decisions [11].

This study aimed to develop a predictive model to estimate the individual risk of MRS infection in patients with pneumonia and build a dynamic nomogram to facilitate clinical use.

## 2. Materials and Methods

### 2.1. Study Population and Design

This observational study enrolled pneumonia patients who were admitted to Cho Ray Hospital from March 2021 to March 2023. The inclusion criteria included (1) being over 18 years of age, (2) having a confirmed diagnosis of pneumonia, and (3) having MRS risks according to ATS recommendations. These included prior intravenous antibiotic use within 90 days, severe pneumonia, especially if intubated, and shock sepsis. Patients with COVID-19 infections or poor-quality specimens were excluded.

### 2.2. Data Collection

Baseline demographics included age, sex, comorbidities, previous infection with MRS, severity of pneumonia, clinical symptoms, and characteristics of chest X-rays or computed tomography scans. All were recorded when the patients had pneumonia.

### 2.3. Definition of Variables

Pneumonia was identified by the presence of a new or worsened lung infiltration on a chest X-ray and meeting at least two of the following criteria: temperature above 38 °C, leukocytosis (≥12,000 WBC/mm^3^) or leukopenia (≤4000/mm^3^), and purulent sputum [2,13].

Pneumonia was classified as hospital-acquired pneumonia (HAP), ventilator-associated pneumonia (VAP), and community-acquired pneumonia (CAP) [13,14]. 

Comorbid illnesses were identified by the Charlson comorbidity index and using ICD-10 cm codes [15]. The method to calculate the CCI score has already been mentioned elsewhere [14,15]. 

The diagnosis of MRS pneumonia was made based on clinical characteristics, laboratory testing, imaging findings, microbiological tests, and treatment outcomes. MRS (MRSA and MRCoNS) that was detected from blood or a respiratory source (such as sputum, endotracheal aspirate, bronchoalveolar lavage, or pleura) through culture and/or real-time PCR [2,13] are deemed clinically significant infection.

### 2.4. Statistical Analysis

Continuous variables were summarized in means and standard deviations if normally distributed, or median and percentile range if otherwise. Measurement data were analyzed by *t* tests (normal distribution) and Mann–Whitney U tests (non-normal distribution). Categorical variables are shown as numbers and percentages for comparison using the χ^2^ test. 

### 2.5. Predictive Model Development and Validation

The initial dataset was used for model development. Stepwise selection and Bayesian model averaging (BMA) were applied to build a prediction model. Covariates were chosen based on p values and Bayesian information criterion [16,17].

For stepwise selection, univariable logistic regression was used in the dataset to identify the independent risk factors for MRS infection. Three approaches were considered for the final model. Models 1 and 2 included all significant predictors from univariable logistic regression with significant p-value levels below 0.1 and 0.05, respectively. In contrast, model 3 retained only those that were significant (*p*-value < 0.1) in the multivariable logistic regression of model 1 [16]. 

In BMA approach, 5 models with the lowest Bayesian information criterion were considered [16,17]. 

The model’s discrimination for MRS infection was evaluated using the receiver operating characteristic (ROC) curve and the area under the curve (AUC). With the threshold, we chose a high sensitivity value, and the net benefit of using the MRS model criteria was significantly different for all treatments. 

The calibration curve determined the model’s goodness of fit. Decision curve analysis and a clinical impact curve were employed to assess the clinical impact of the model. The model with the best AUC value in the dataset was chosen as an optimal model, which was then translated to a static and a web-based nomogram. 

A total of 1000 bootstrap samples (with replacement) were employed to evaluate the model. The potential impact of utilizing the MRS model for screening MRS testing was measured. Initially, the infected case rate was used as a testing threshold for the MRS model to determine the expected infection risk. Within the bootstrap samples, the AUC and sensitivity of the model were calculated. The net benefit of employing the MRS model criteria was compared to that of treating all patients [18].

All statistical analyses were performed using the R Studio program version 4.2.3. R software (rms, tidyverse, caret, and BMA package) to build the nomogram models. Then, DynNom and Shiny packages were used to build a web application. 

## 3. Results

### 3.1. Baseline Characteristics of the Study Population

**Figure 1:**
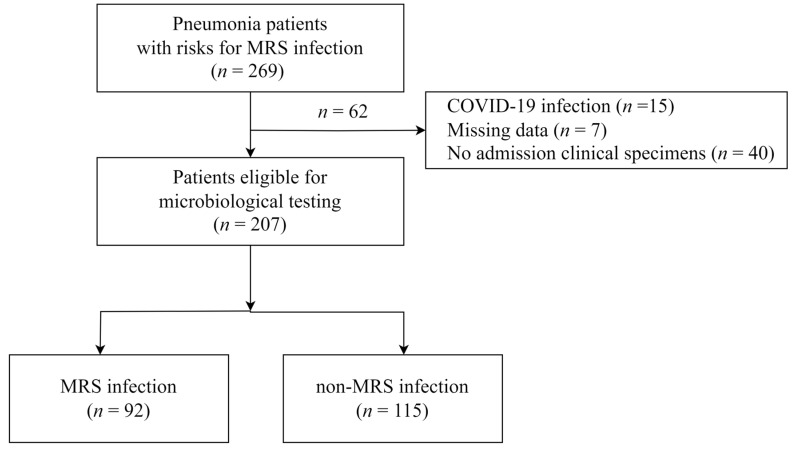
Flow diagram of the study. MRS—methicillin-resistant *Staphylococcus* spp.

The study included 207 patients with 92 (44.44%) MRS infections and 115 (55.56%) non-MRS infections (Figure 1). In this study, 207 eligible subjects, including 113 (54.59%) HAP, 31 (14.98%) VAP, and 63 (30.43%) CAP with risk factors for MDR pathogens, were enrolled. There were 146 (70.53%) males and 61 (29.47%) females. The mean age was 60.47 ± 16.03 years (Table 1).

### 3.2. Predictive Model Development and Validation

All baseline characteristics in the dataset were included in the univariable logistic regression analysis. At the a priori significance level *p*-value of 0.1, prior intravenous antibiotic use within 90 days, severity of pneumonia, respiratory failure, tracheostomy or endotracheal tube, urinary catheterization, central venous catheterization, nasogastric intubation, skin infection, and pneumatoceles were associated with the risk of MRS infection (Table 1). Furthermore, skin infection and pneumatoceles were significantly associated with the risk of MRS infection in the multivariable logistic regression analysis (Table 2).

**Figure 2:**
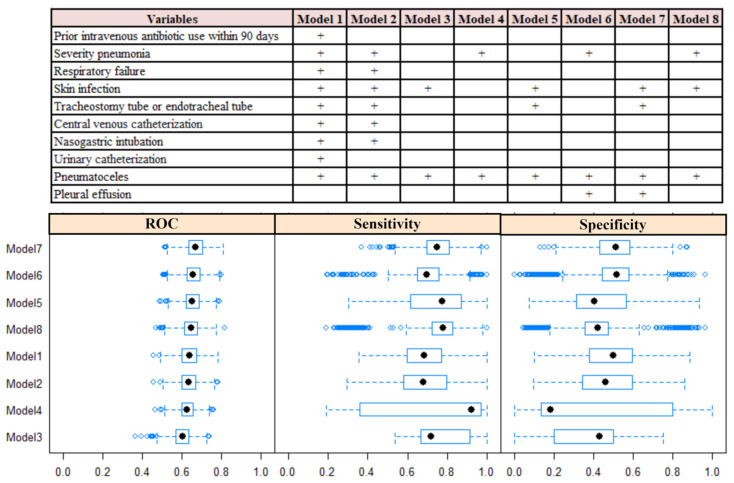
Variables, receiver operating characteristic, sensitivity, and specificity values of the predictive models. ROC—receiver operating characteristic; sens—sensitivity; spec—specificity.

**Figure 3:**
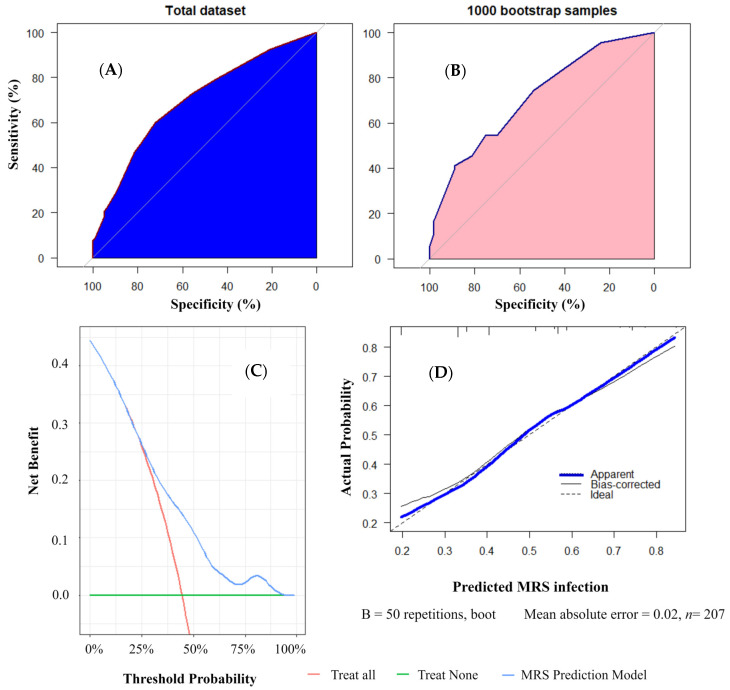
Receiver operating characteristic curve of the total dataset (**A**) and 1000 bootstrap samples (**B**), net benefit (**C**), and calibration curve (**D**) of model 7. MRS—methicillin-resistant *Staphylococcus* spp.

**Figure 4:**
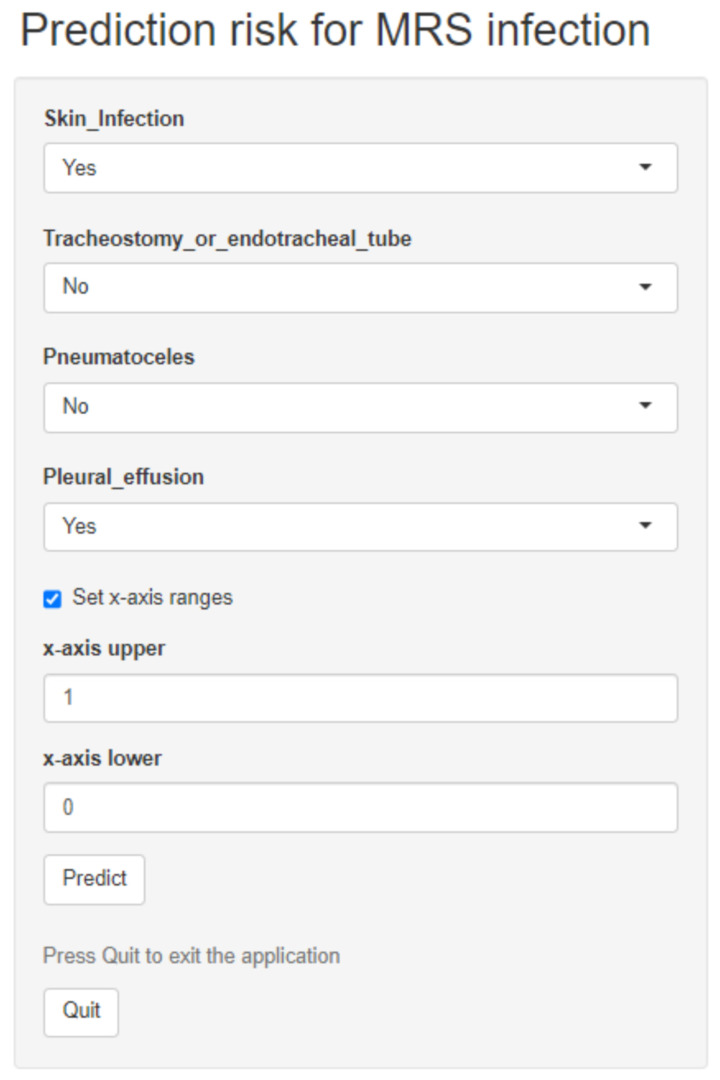
A dynamic nomogram to predict the risk of methicillin-resistant *Staphylococcal* infection in patients with pneumonia. MRS—Methicillin-resistant *Staphylococcus* spp.

As shown in Figure 2, model 7 with four predictors (tracheostomies and endotracheal tube, skin infection, pleural effusions, and pneumatoceles) outperformed other models with regard to their ROC and sensitivity values in the dataset. ROC analyses showed an area under the curve of 0.70 (0.63–0.77) and 0.71 (0.64–0.78) (Figure 3A,B) in the two datasets. As shown in Figure 3C, the net benefit of the decision curve for the nomogram was higher, suggesting that our nomogram could improve clinical prediction. Additionally, the calibration curve revealed a close alignment between the predicted probability and the actual estimates (Mean Absolute Error = 0.02) (Figure 3D), affirming the model’s goodness of fit. Furthermore, at the threshold of 0.3, the model showed a sensitivity of 92.39% and 91.11% in the dataset and 1000 bootstrap samples, respectively (Table 3). With this threshold, we reduced24 patients who did not need to receive anti-MRSA therapy (Table S1). At the threshold of 0.8, the model showed a specificity of 100% and 95.73% in the order of samples given (Table 3). The nomogram was thereby developed based on model 7 and is available at “https://bvv2023.shinyapps.io/MRSapp/ (accessed on 11 March 2024)” (Figure 4). 

## 4. Discussion

In this study, prior intravenous antibiotic use within 90 days, severity of pneumonia, respiratory failure, tracheostomy or endotracheal tube, urinary catheterization, central venous catheterization, nasogastric intubation, skin infection, and pneumatoceles were associated with MRS pneumonia at a *p*-value of below 0.1. These risk factors for MRS infection are in line with the current literature [7,8]. According to the American Thoracic Society (ATS), hospitalization history, especially frequent hospital admissions and prolonged hospital stays, is independently associated with MRSA infection [19]. Additionally, colonization and infection of the skin by *Staphylococcus* spp. also increase the risk of pneumonia and bloodstream infections [20]. Patients with tracheostomies and endotracheal tubes were vulnerable to biofilm-producing and highly resistant bacteria quickly colonizing the surfaces of the tracheostomy tubes. A retrospective study of intensive care units over 10 years revealed that *Staphylococcus* spp., including CoNS and *S. aureus*, were 1 of 14 common microorganisms isolated from tracheostomy tubes [21]. Furthermore, pleural effusions, pneumatoceles, and pneumothoraces are also common findings in radiological investigations and a computed tomography scan in pneumonia caused by *S. aureus* [22]. 

Scoring systems and nomograms are necessary and convenient for predicting MRS infection. A cohort study derived a risk score for MRSA infection from 5975 patients, with 14% having MRSA infections. The score consisted of eight variables and a possible total score of 10. The MRSA infection probability was lower by 10% at a score of 0 to 1 and could be above 30% when the score was 6 or greater [7]. Another retrospective study showed that a history of MRSA infection (OR 5.6, 95% CI 1.56–20.63) and osteomyelitis (OR 2.5, 95% CI 1.00–6.79) was linked to a higher chance of MRSA isolation (C statistic of 0.7). Previous work also introduced a predictive nomogram with moderate to good discrimination for MRSA infection in patients with infected foot ulcers [23]. In this study, we developed a model and used the bootstrap method to validate the nomogram with an AUC value of 0.70 and a sensitivity of 92.39% at a threshold of 0.3 in the dataset (Figure 2 and Figure 3A,B and Table 3). Our model demonstrated good calibration, as substantial agreement between the observed and predicted probability curves was found in the calibration analysis (Figure 3C,D). 

Early diagnosis and timely treatment play a crucial role in MRS pneumonia management. MRSA bacteremia substantially increases hospital costs, as patients with this infection required an additional 12,818 (95% CI 7246–19,966) hospital bed days, which consequently cost the hospitals an extra USD 24,366,741 (95% CI USD 13,774,548–37,954,686) per year [24]. Therefore, ATS has recommended covering MRSA early in patients who have had prior intravenous antibiotic use within 90 days, severe pneumonia, or a previous infection with MRSA [9,13]. Regarding the pathogen’s prevalence, however, among 3562 cases, only 5.2% were positive for MRSA. The recommended empirical anti-MRSA coverage over a low infection prevalence might render up to 94.8% of patients overtreated [25]. Among 88,605 patients admitted to the hospital, empirical anti-MRSA therapy was given to 33,632 individuals (38%). When compared to standard treatment alone, the addition of empirical anti-MRSA therapy alongside standard therapy showed a significant association with increased adjusted risks of death (95% CI 1.3–1.5), kidney injury (95% CI 1.3–1.5), secondary Clostridium difficile infections (95% CI 1.3–1.9), vancomycin-resistant Enterococcus spp. infections (95% CI 1.0–2.3), and secondary Gram-negative rod infections (95% CI 1.2–1.8) [26]. Furthermore, CoNS are one of the major opportunistic pathogens, and there are fewer reports of these pathogens in pneumonia; however, it was indicated that the antibiotic resistance, failed rate of treatment, and 30-day mortality for CoNS are higher than those for other organisms [27]. Obviously, a pathogen-specific treatment should be favored over cover therapies in view of treatment costs and adverse effects such as kidney injury, secondary infection, and antibiotic resistance. Our model had a good net benefit, as shown in the decision curve, implying greater benefit when applied to guide treatment (Figure 3C). In patients with pneumonia who had prior intravenous antibiotic use within 90 days, severe pneumonia, or previous MRS infection plus a positive prediction of our MRS app, we reduced 24 cases of pneumonia with coverage treatment for MRS of the ATS recommendation (Table S1).

Our research should bring some benefits to clinical practice. ATS and BTS recommendations helped to determine the best empiric therapy for MRS infection, but imbalances between cost effectiveness, complications, and positive outcome have been the subjects of many debates, so our model could serve as a screening tool plus recommendations. In addition, with a high sensitivity, a good AUC, a high net benefit in decision curve analysis, and good calibration, our model represents a useful and effective prediction tool. The model was made available as a web-based interactive tool to ease clinical use and enhance its accessibility. The number of MRS cases in our research was sufficient to build a model that responded to at least 200 observations [28] and complied with the rules of thumb (1:10) [29] or Claudia Beleites (5–25 independent samples per class) [30] and other predictive research [31]. Furthermore, the quality of the model is measured based on two factors, including accuracy and precision of estimation. In our research, we used many methods to select variables for building and comparing models and used bootstrapping to estimate the precision of the unknown population characteristics.

This study may be subject to limitations that warrant consideration. Firstly, the research was conducted at a single center, which inherently restricts the generalizability of the model. Secondly, the sample size was relatively small in relation to the number of covariates included, potentially leading to an overfitted model. It is crucial to conduct additional validation studies in diverse settings to corroborate the findings and enhance the robustness of the model.

## 5. Conclusions

This study reaffirmed the significance of prior intravenous antibiotic use within 90 days or severe pneumonia as important risk factors for MRS pneumonia. The nomogram model demonstrated commendable performance in predicting MRS infection. Exploring the potential benefits of implementing this model for early administration of MRS antibiotics merits further investigation.

## Figures and Tables

**Table 1:** Demographics and clinical characteristics of the study.

Variables, *n* (%)	Total	Non-MRS Infection (*n* = 115)	MRS Infection *(n* = 92)	*p* Value
Sex				
Female	61 (29.47)	33 (28.70)	28 (30.43)	0.905
Male	146 (70.53)	82 (71.30)	64 (69.57)	
Comorbidities				
Myocardial infartion	4 (1.93)	1 (0.87)	3 (3.26)	0.463
Congestive heart failure	21 (10.14)	9 (7.83)	12 (13.04)	0.316
Peripheral vascular disease	3 (1.45)	2 (1.74)	1 (1.09)	1.000
Solid tumor	6 (2.90)	1 (0.87)	5 (5.43)	0.126
Cerebrovascular disease	7 (3.38)	5 (4.35)	2 (2.17)	0.636
Ulcer disease	13 (6.28)	6 (5.22)	7 (7.61)	0.677
Diabetes	31 (14.98)	16 (13.91)	15 (16.30)	0.777
Chronic obstructive pulmonary disease	33 (15.94)	19 (16.52)	14 (15.22)	0.949
Moderate or severe renal disease	37 (17.87)	22 (19.13)	15 (16.30)	0.730
Diabetes with complications	42 (20.29)	20 (17.39)	22 (23.91)	0.324
Hemiplegia	18 (8.70)	11 (9.57)	7 (7.61)	0.804
Moderate or severe liver disease	16 (7.73)	8 (6.96)	8 (8.70)	0.839
Metastatic solid tumor	13 (6.28)	9 (7.83)	4 (4.35)	0.461
AIDS	4 (1.93)	1 (0.87)	3 (3.26)	0.463
Previously infected with MRSA	6 (2.90)	1 (0.87)	5 (5.43)	0.126
Prior intravenous antibiotic use within 90 days	153 (73.91)	79 (68.70)	74 (80.43)	0.080
After surgery	25 (12.08)	13 (11.30)	12 (13.04)	0.867
Immunodeficiency	38 (18.36)	21 (18.26)	17 (18.48)	1.000
Severity of pneumonia	148 (71.50)	72 (62.61)	76 (82.61)	0.003
Fever	17 (8.21)	9 (7.83)	8 (8.70)	1.000
Sputum	197 (95.17)	112 (97.39)	85 (92.39)	0.180
Dyspnea	179 (86.47)	95 (82.61)	84 (91.30)	0.107
Chest pain	38 (18.36)	20 (17.39)	18 (19.57)	0.825
Skin infection	67 (32.37)	29 (25.22)	38 (41.30)	0.021
Respiratory failure	151 (72.95)	76 (66.09)	75 (81.52)	0.020
Sedative drug use	23 (11.11)	12 (10.43)	11 (11.96)	0.902
Shock	36 (17.39)	20 (17.39)	16 (17.39)	1.000
Dialysis	13 (6.28)	5 (4.35)	8 (8.70)	0.321
Tracheostomy tube or endotracheal tube	88 (42.51)	39 (33.91)	49 (53.26)	0.008
Central venous catheterization	87 (42.03)	40 (34.78)	47 (51.09)	0.026
Urinary catheterization	87 (42.03)	41 (35.65)	46 (50.00)	0.053
Nasogastric intubation	90 (43.48)	41 (35.65)	49 (53.26)	0.016
Pleural drainage	15 (7.25)	5 (4.35)	10 (10.87)	0.126
Insulin therapy	52 (25.12)	25 (21.74)	27 (29.35)	0.274
Consolidation	201 (97.10)	113 (98.26)	88 (95.65)	0.487
Pulmonary cavities	20 (9.66)	13 (11.30)	7 (7.61)	0.511
Pneumatoceles	21 (10.14)	6 (5.22)	15 (16.30)	0.017
Pleural effusion	101 (48.79)	50 (43.48)	51 (55.43)	0.116
Levels of BMI				
Underweight	55 (26.57)	31 (26.96)	24 (26.09)	0.526
Normal range	95 (45.89)	55 (47.83)	40 (43.48)	
Overweight	32 (15.46)	14 (12.17)	18 (19.57)	
Obese	25 (12.08)	15 (13.04)	10 (10.87)	
Types of nosocomial pneumonia				
HAP	113 (54.59)	67 (58.26)	46 (50.00)	0.493
VAP	31 (14.98)	16 (13.91)	15 (16.30)	
CAP	63 (30.43)	32 (27.83)	31 (33.70)	
Levels of age				
Under 60	91 (43.96)	52 (45.22)	39 (42.39)	0.790
Above 60	116 (56.04)	63 (54.78)	53 (57.61)	
Age (mean [SD])	60.47 ± 16.03	60.23 ± 15.85	60.78 ± 16.32	0.805
Commodity Channel Index	2 (0–8.85)	2 (0–9)	2 (0–6.73)	0.982

Data are presented as the mean and SD or number and proportion of participants in each diagnosis category (%) where data were available. MRS—methicillin-resistant *Staphylococcus* spp.; AIDS—acquired immune deficiency syndrome; MRSA—methicillin-resistant *Staphylococcus aureus*; BMI—body mass index; HAP—hospital-acquired pneumonia; VAP—ventilator-associated pneumonia; CAP, community-acquired pneumonia; SD—standard deviation.

**Table 2:** Multivariable logistic regression analysis of the dataset.

Variables, *n* (%)	OR	95%Cl	*p*-Value
Prior intravenous antibiotic use within 90 days	1.71	0.83–3.65	0.153
Severity of pneumonia	1.72	0.65–4.71	0.279
Skin infection	1.89	1.00–3.62	0.052
Respiratory failure	1.27	0.46–3.45	0.643
Tracheostomy tube or endotracheal tube	1.26	0.50–3.19	0.626
Central venous catheterization	1.32	0.47–3.72	0.594
Nasogastric intubation	3.12	0.66–15.92	0.154
Urinary catheterization	0.36	0.07–1.49	0.175
Pneumatoceles	5.11	1.81–16.23	0.003

OR—odds ratio; CI—Confidence Interval.

**Table 3:** Sensitivity and specificity of the dataset and 1000 bootstrap samples to different threshold values.

Threshold	Dataset		Bootstrap	
	Sensitivity	Specificity	Sensitivity	Specificity
0.1	-	-	-	-
0.15	-	-	-	-
0.2	92.39	20.87	91.11	19.66
0.25	92.39	20.87	91.11	19.66
0.3	92.39	20.87	91.11	19.66
0.35	72.83	55.65	63.33	47.86
0.4	59.78	72.17	46.67	61.54
0.45	59.78	72.17	46.67	61.54
0.5	59.78	72.17	46.67	61.54
0.55	51.09	78.26	38.89	68.38
0.6	21.74	93.91	15.56	88.89
0.65	21.74	93.91	15.56	88.89
0.7	21.74	93.91	15.56	88.89
0.75	8.70	99.13	3.33	94.87
0.8	7.61	100.0	2.22	95.73
0.85	4.35	100.0	1.11	97.44
0.9	1.09	100.0	0	99.15
0.95	-	-	-	-
1	-	-	-	-

## Data Availability

The datasets used and/or analyzed during the current study are available from the corresponding author on reasonable request.

## Supplementary Materials

The following supporting information can be downloaded at https://www.mdpi.com/article/10.3390/diagnostics14060633/s1, Table S1. Clinical attributes of our predictive model.
