# Supplementary material for: A Web-Based Dynamic Nomogram to Predict the Risk of Methicillin-Resistant Staphylococcal Infection in Patients with Pneumonia
Source: Diagnostics (Basel). 2024 Mar 16;14(6):633. doi: 10.3390/diagnostics14060633 (PMC10969305; doi:10.3390/diagnostics14060633)
Supplement: Supplementary file 1 [file diagnostics-14-00633-s001.zip › diagnostics-2890134-supplementary.pdf]

**Table S1. Clinical attributes of our predictive model.**

| | | MRS infection | |
|---|---|---|---|
| | | Positive | Negative |
| Our predictive model | Positive | 85 | 91 |
| | Negative | 7 | 24 |

MRS—Methicillin-resistant Staphylococcus spp.
